# The Source and Evolutionary History of a Microbial Contaminant Identified Through Soil Metagenomic Analysis

**DOI:** 10.1128/mBio.01969-16

**Published:** 2017-02-21

**Authors:** Matthew R. Olm, Cristina N. Butterfield, Alex Copeland, T. Christian Boles, Brian C. Thomas, Jillian F. Banfield

**Affiliations:** a University of California, Berkeley, California, USA; b Joint Genome Institute, Walnut Creek, California, USA; c Sage Science, Inc., Beverly, Massachusetts, USA; Michigan State University; California Institute of Technology/HHMI

## Abstract

In this study, strain-resolved metagenomics was used to solve a mystery. A 6.4-Mbp complete closed genome was recovered from a soil metagenome and found to be astonishingly similar to that of *Delftia acidovorans* SPH-1, which was isolated in Germany a decade ago. It was suspected that this organism was not native to the soil sample because it lacked the diversity that is characteristic of other soil organisms; this suspicion was confirmed when PCR testing failed to detect the bacterium in the original soil samples. *D. acidovorans* was also identified in 16 previously published metagenomes from multiple environments, but detailed-scale single nucleotide polymorphism analysis grouped these into five distinct clades. All of the strains indicated as contaminants fell into one clade. Fragment length anomalies were identified in paired reads mapping to the contaminant clade genotypes only. This finding was used to establish that the DNA was present in specific size selection reagents used during sequencing. Ultimately, the source of the contaminant was identified as bacterial biofilms growing in tubing. On the basis of direct measurement of the rate of fixation of mutations across the period of time in which contamination was occurring, we estimated the time of separation of the contaminant strain from the genomically sequenced ancestral population within a factor of 2. This research serves as a case study of high-resolution microbial forensics and strain tracking accomplished through metagenomics-based comparative genomics. The specific case reported here is unusual in that the study was conducted in the background of a soil metagenome and the conclusions were confirmed by independent methods.

## IMPORTANCE

It is often important to determine the source of a microbial strain. Examples include tracking a bacterium linked to a disease epidemic, contaminating the food supply, or used in bioterrorism. Strain identification and tracking are generally approached by using cultivation-based or relatively nonspecific gene fingerprinting methods. Genomic methods have the ability to distinguish strains, but this approach typically has been restricted to isolates or relatively low-complexity communities. We demonstrate that strain-resolved metagenomics can be applied to extremely complex soil samples. We genotypically defined a soil-associated bacterium and identified it as a contaminant. By linking together snapshots of the bacterial genome over time, it was possible to estimate how long the contaminant had been diverging from a likely source population. The results are congruent with the derivation of the bacterium from a strain isolated in Germany and sequenced a decade ago and highlight the utility of metagenomics in strain tracking.

## INTRODUCTION

Microbial strains of the same species can have very different traits, including virulence and drug resistance (1–3); thus, tracking of specific strain populations is important in a number of different contexts. Microbial source tracking (MST) via quantitative PCR is routinely used to determine the source of fecal bacteria in environmental waters (4) and, in some cases, can discriminate between the fecal profiles of different types of animals (5). Tracing pathogenic strains within hospitals via sequencing of isolated strains can uncover vectors of nosocomial infections (6), and larger-scale studies have improved our understanding of the intercontinental spread of pathogens (7). The forensic investigation launched following the 2001* B. anthracis* bioterrorism attack has been called “one of the largest and most complex in the history of law enforcement” (8). Significant effort since has been invested to develop new methods, including clustered regularly interspaced short palindromic repeat(s) (CRISPR)-Cas analysis (9), to deploy in the case of future bioterrorism events (10).

A key component of microbial forensics is strain typing. Strain resolution is essential to trace the spread of a population, and more sensitive strain typing methods can provide higher-resolution transmission maps. The most common methods identify unique (but small) markers of the microbial population’s genomic DNA sequence and take advantage of the fact that random mutations develop in all growing populations. Examples of methods to identify these mutations include restriction endonuclease analysis, pulsed-field gel electrophoresis, ribotyping, and multilocus sequencing typing (11, 12). Decreasing sequencing costs have also allowed an increasing number of studies to take advantage of genome sequencing, the “gold standard” of microbial typing (6). This approach can discriminate between microbial populations that differ by even a single nucleotide, but typically this requires culturing of the organism before DNA extraction, and this is not feasible in all cases. Metagenomics, on the other hand, has the potential to trace and characterize virulent strains without cultivation and could be used to detect strains of interest in environmental samples (13).

The National Academy of Sciences stated that metagenomics “will bring about a transformation in biology, medicine, ecology, and biotechnology that may be as profound as that initiated by the invention of the microscope” (14). In metagenomics, shotgun sequencing of DNA extracted directly from environmental samples allows characterization of microbes without the need for cultivation. Assembly and binning of short metagenomic reads can yield hundreds of genomes from metagenomic samples (15–18). However, sequencing projects can be contaminated with exogenous DNA (19). Significant efforts have been made to determine where these contaminants originate (20), but precise sources of contaminant sequences are seldom identified. The mystery of determining the source of contaminant DNA in the background of a complex metagenomic sample represents a useful test case for genomics-based microbial forensics.

## RESULTS

### Recovery of a complete *Delftia acidovorans* genome from soil.

Soil samples were collected from two locations in the Angelo Coast Range Reserve in northern California. A meadow within the reserve was sampled at two soil depths (20 and 40 cm) over a 6-week period, and a ridge nearby was sampled at six depths (15 to 115 cm, including both soil and underlying weathered shale) during one sampling event. The sites are located within the Eel Critical Zone Observatory. Metagenomic DNA was extracted from all of the samples, and between 15.7 and 44.0 Gbp of Illumina shotgun paired-end sequencing was generated for each sample. In total, 0.4 Tbp of sequence data was generated (see Table S1 in the supplemental material). Shannon diversity was calculated for all of the samples by using ribosomal protein S3 (rpS3) genes. The mean alpha diversity of the soil collected in this study was 4.65 (standard deviation, 0.28), similar to that of many previously studied soils (21–23) (see Table S1).

10.1128/mBio.01969-16.4 TABLE S1  Project information (sequencing depth, naming, descriptions, etc.). Download TABLE S1, XLSX file, 0.04 MB. Copyright © 2017 Olm et al. 2017 Olm et al. This content is distributed under the terms of the Creative Commons Attribution 4.0 International license.

An initial binning analysis revealed genome fragments that were profiled as deriving from *D. acidovorans* populations in six samples (other genomes were reported by Butterfield et al. [24]). We reconstructed a 6.41-Mbp high-quality draft genome of *D. acidovorans* consisting of 53 scaffolds ranging in length from 6 to 500 kbp. The genome was further curated by read mapping to fill scaffolding gaps and extend and join the contigs. The resulting 16 contigs could be ordered and oriented to the previously sequenced *D. acidovorans* SPH-1 genome (GenBank accession no. NC_010002.1). The contigs spanned 97.95% of the SPH-1 genome, with an average nucleotide identity (ANI) of 99.99% (25). Because of the remarkably high nucleotide similarity between our recovered genome and that of strain SPH-1, we further curated the 16 contigs into one circular genome by using the SPH-1 genome to identify reads in our metagenomic data set that filled the gaps. When visualized with the software package Geneious (26), single nucleotide polymorphisms (SNPs) and insertions and deletions relative to the isolate genome sequence were easily identified (see Fig. S1). The circular closed genome represents the bacterial strain *D. acidovorans* ANG1.

10.1128/mBio.01969-16.1 FIG S1  Differences between genomes and reads are readily identified. Shown are reads mapping from sample SNNY to the *D. acidovorans* SPH-1 genome, revealing a transposon present in the reference genome that is absent from the project reads. Download FIG S1, PDF file, 0.4 MB. Copyright © 2017 Olm et al. 2017 Olm et al. This content is distributed under the terms of the Creative Commons Attribution 4.0 International license.

### Identification of *D. acidovorans* as a contaminant.

**FIG 1:**
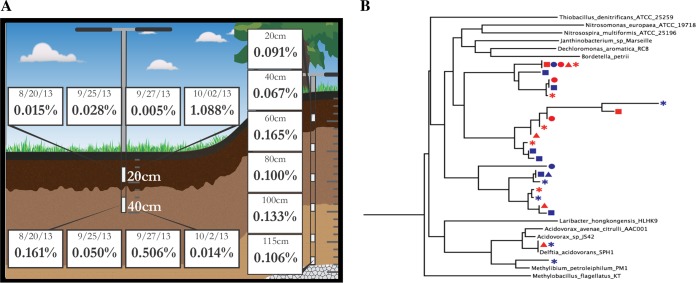
*D. acidovorans* is present in all of the soil and sediment samples sequenced, but the population structure is distinct from that of other bacteria. (A) The percentage of the reads from each sample that map to the *D. acidovorans* ANG1 genome. (B) An rps3 phylogenetic tree for a typical bacterial group (*Betaproteobacteria*) showing a dandelion-like pattern of strain diversity that is dissimilar from that of *D. acidovorans* (only the full-length assembled sequences are shown). Branches ending with a taxonomic identification are reference sequences, and the soil sequences are indicated by colored shapes representing their soil sample depth and time of origin around the first rainfall from August to October 2013 (10 to 20 cm, blue; 30 to 40 cm, red) before (squares) and after the rain events (4 days after the second rain, circles; 6 days after the second rain, triangles; 2 days after the second rain, asterisks).

On the basis of metagenomic read mapping, *D. acidovorans* ANG1 was detected in all 14 samples from two sites. No pattern between the sampling location and the abundance of *D. acidovorans* could be detected (Fig. 1A). On the basis of mapping of reads to the ANG1 genome, it was determined that the *D. acidovorans* reads in all of the samples derived from a single genotype (see Table S1). This result contrasts with our general findings from soil metagenomics, which typically indicate the presence of multiple closely related strains. For example, a phylogenetic tree constructed with rpS3 gene sequences shows dandelion-like strain diversity patterns in betaproteobacteria from the same samples (Fig. 1B). The lack of strain diversity in *D. acidovorans*, in combination with the high similarity of the *D. acidovorans* genome to that of the SPH-1 strain isolated years earlier, raised the possibility that *D. acidovorans* ANG1 was not a native member of the soil community. A PCR test with primers designed to target random regions of the *D. acidovorans* genome failed to detect DNA from this bacterium in the original DNA extracted from the samples (Fig. S2). This indicated that the DNA was probably introduced into our samples during sequencing. Moreover, the detection of the same genotype in samples sequenced at different times suggested that *D. acidovorans* was a persistent contaminant at this facility.

10.1128/mBio.01969-16.2 FIG S2  PCR testing confirms introduction of* D. acidovorans* DNA after DNA extraction. Primers binding to a random region of the* D. acidovorans* genome fail to amplify *D. acidovorans* DNA from a retained aliquot of soil sample SNNY, providing evidence that *D. acidovorans* was not present in the original soil sample or introduced during DNA extraction. Download FIG S2, PDF file, 0.5 MB. Copyright © 2017 Olm et al. 2017 Olm et al. This content is distributed under the terms of the Creative Commons Attribution 4.0 International license.

### Source tracking of contaminant *D. acidovorans.*

**FIG 2:**
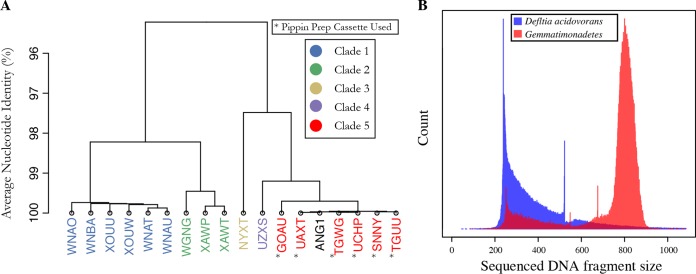
*D. acidovorans* contamination originates from library size selection cassettes. (A) Hierarchical clustering of *D. acidovorans* strains present in shotgun sequencing projects sequenced at the same facility as soil metagenomes in this study, on the basis of shared SNP frequencies. Samples prepared with Pippin Size Selection Cassettes (Sage Science) are marked with asterisks. Sequences of *D. acidovorans* in those samples form a monophyletic group that includes the ANG1 genome. (B) Histogram of sequenced DNA fragment sizes of metagenomic reads mapping to the recovered *D. acidovorans* genome and the second most abundant recovered genome in the sample, from a *Gemmatimonadetes* bacterium. The placements of reads mapping to the *Gemmatimonadetes* genome indicate the expected 800-bp fragment length, yet reads mapping to *D. acidovorans* show a different and skewed profile, consistent with the introduction of DNA after size selection.

To further investigate the possibility that DNA from *D. acidovorans* ANG1 was introduced in the sequencing facility, we screened 43 publicly available metagenomes sequenced at this facility between June 2012 and January 2015. Seventeen of these projects had ≥20% of the *D. acidovorans* genome present, with coverage of >5× (see Table S2). Multiple strains were present in these projects, on the basis of analysis of the patterns of SNPs relative to the *D. acidovorans* ANG1 genome (Fig. 2A; see Table S3). Six projects, including three plant genomes and our soil samples, contained sequences that clustered with the *D. acidovorans* ANG1 genome. We refer to this as the contaminant clade and hypothesized that some reagent in the sequencing pipeline may have been the source of the *D. acidovorans* DNA in these six projects. Records provided by the sequencing facility revealed that each project containing the *D. acidovorans* ANG1 contaminant clade used a Pippin Prep size selection cassette. All of the other projects analyzed did not use these cassettes (Fig. 2A).

10.1128/mBio.01969-16.5 TABLE S2  JGI projects and how much *Delftia* DNA they contain. Download TABLE S2, XLSX file, 0.04 MB. Copyright © 2017 Olm et al. 2017 Olm et al. This content is distributed under the terms of the Creative Commons Attribution 4.0 International license.

10.1128/mBio.01969-16.6 TABLE S3  Pairwise ANI between all contaminant clade projects. Download TABLE S3, XLSX file, 0.1 MB. Copyright © 2017 Olm et al. 2017 Olm et al. This content is distributed under the terms of the Creative Commons Attribution 4.0 International license.

To test the hypothesis that the Pippin Prep size selection cassettes were the source of *D. acidovorans* ANG1 contamination, we analyzed the insert sizes of reads from all of the projects mapped to the ANG1 genome. If the contaminant DNA was introduced from the library preparation cassette, it should have a more random fragment size profile, and thus insert size profile, than the tight size profile generated during library preparation. A histogram of reads mapping to the *D. acidovorans* ANG1 genome from a sample exhibiting substantial contamination is shown in Fig. 2B, along with a histogram of reads mapping to the genome of a *Gemmatimonadetes* strain known to be native to the same sample. The fragment size of paired reads mapped to the *Gemmatimonadetes* genome was ~800 bp. In contrast, read mapping to *D. acidovorans* indicates that many of the sequencing reads were generated from fragments of around 250 bp (in these cases, the 250-bp *D. acidovorans* reads overlap completely). However, the *D. acidovorans* peak is strongly skewed and some of the fragments were >1,000 bp in length. These observations indicate that the contaminant DNA was present in the gel and/or buffer used for size selection. All of the projects that contained *D. acidovorans* from the contaminant clade had similar insert size histograms, whereas projects with other *D. acidovorans* clades had normal insert sizes.

**FIG 3:**
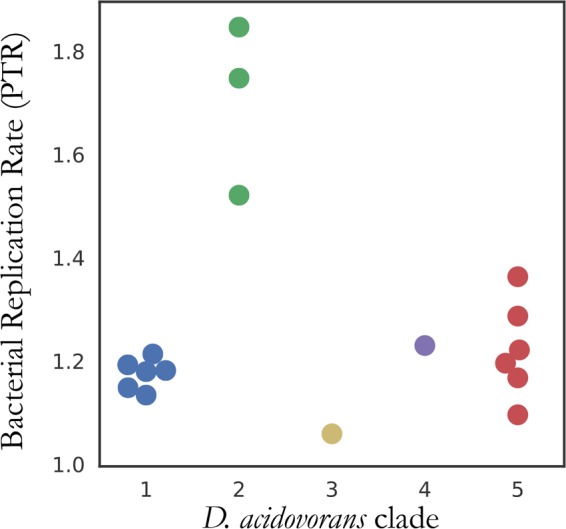
*D. acidovorans* strains native to a bioreactor have distinct replication rates. Clades of similar *D. acidovorans* genomes were clustered on the basis of shared SNP frequencies (Fig. 2A). The PTRs are significantly higher (*P* = 0.005) for the strains growing in a thiocyanate bioreactor (clade 2) than for strains growing in the other environments. As the other clades are likely made up of contaminants (see text), this observation supports the conclusion that the bioreactor strains are active community members and not contaminants.

Sage Science, the producer of the Pippin size selection cassettes, was contacted regarding our observations. They explained that bacterial biofilms were present in tubing that delivered buffer to the cassettes and stated that the problem was corrected in 2013. All six projects that contain the contaminant clade used cassettes made prior to the correction, and libraries made with cassettes produced after Sage Science revised its manufacturing process to keep buffer tubing bacterium free did not reveal *D. acidovorans* ANG1 contamination (data not shown). The sequencing facility continued to detect *D. acidovorans* in samples sequenced after Sage Science corrected the problem and so concluded that sequences were not from the Pippin cassettes. We show here that the newly detected sequences were from the other clades, either strains actually present in the samples or contaminants from a different source. For example, clade 1 is associated with six metagenomes of the upper troposphere and clade 2 is associated with three metagenomes of thiocyanate bioreactor communities (see Table S2). In the case of clade 2, we conclude that *D. acidovorans* was native to the sample because the population was growing rapidly, on the basis of differential coverage at the origin and terminus of replication (peak-to-trough ratios [PTRs]) (full range, 1.5 to 1.9) (27, 28) (see Table S4). In contrast, bacteria of the contaminant clade (clade 5) had consistently low growth rates, based on PTRs between 1.4 and 1.1 (Fig. 3).

10.1128/mBio.01969-16.7 TABLE S4  Bacterial replication rates of *D. acidovorans* populations. Download TABLE S4, XLSX file, 0.03 MB. Copyright © 2017 Olm et al. 2017 Olm et al. This content is distributed under the terms of the Creative Commons Attribution 4.0 International license.

### *In situ* evolution of a bacterial contaminant.

**FIG 4:**
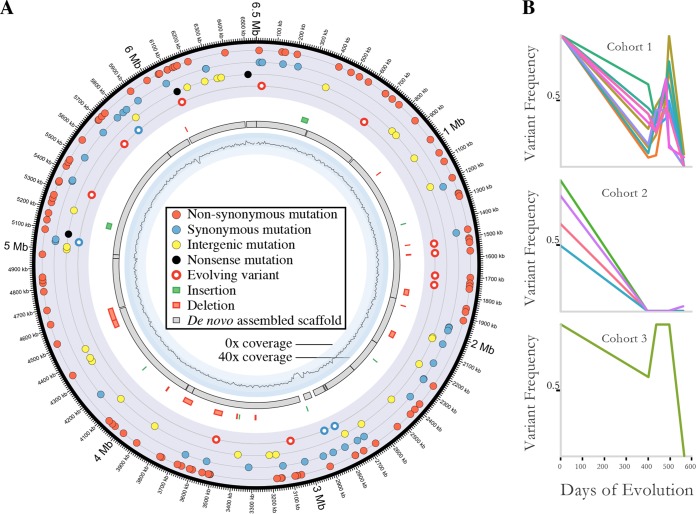
Detailed analysis of *D. acidovorans* sequence variation indicates the presence of subpopulations that are evolving *in situ*. (A) Shown are the locations of all of the differences between the contaminant *D. acidovorans* ANG1 genome recovered in this study and the *D. acidovorans* SPH-1 genome, as well as the alignment of the assembled contigs and coverage of the genome. (B) The frequencies of specific SNPs in samples sequenced at different time points exhibit three distinct patterns suggestive of three subpopulations (cohorts). Abundances fluctuate, but there is an overall progression toward fixation.

Fifteen large (>100-bp) insertions/deletions distinguished the *D. acidovorans* SPH-1 and ANG1 genomes (see Table S5). Four of these were insertions, and 11 were deletions (Fig. 4A). Specifically, two prophages were inserted into the ANG1 genome relative to the SPH-1 genome, but six prophages and five transposons were lost. Another difference involved three adjacent CRISPR repeat-spacer sequences. Finally, we identified a 195-bp insertion that added a pair of transmembrane helices that converted a predicted major facilitator superfamily protein into a predicted transporter that confers drug resistance.

10.1128/mBio.01969-16.8 TABLE S5  All mutations between *D. acidovorans* ANG1 and SPH1 (breseq output). Download TABLE S5, XLSX file, 0.03 MB. Copyright © 2017 Olm et al. 2017 Olm et al. This content is distributed under the terms of the Creative Commons Attribution 4.0 International license.

We documented SNP-based genomic variation over the 1.5-year period to determine whether an evolutionary rate could be measured. A total of 203 SNPs distinguished the ANG1 genome from the *D. acidovorans* SPH-1 isolate genome (see Table S5). Thirty-seven SNPs were very closely spaced on the genome (Fig. 4A) and are statistically unlikely to have formed by individual random mutation events (see Materials and Methods). Specifically, 11 SNPs and four single-base-pair indels occurred in a 64-bp intergenic region upstream of an integrase and 24 SNPs occurred in a 447-bp region within a YD repeat-containing protein possibly involved in carbohydrate binding. We infer that these regions may have been acquired via homologous recombination with a very distinct genotype (<95% nucleotide identity); thus, we did not include them in our analysis of *in situ* genomic change via SNP formation.

We identified 15 SNPs that distinguished the genotypes present in the first and last metagenomes (a separation time of ~1.5 years) and tracked their frequencies over time. Notably, the SNP frequencies cluster into three patterns, consistent with linkage and thus the existence of three subpopulations. Frequencies do not show a simple trend toward fixation, but all SNPs are fixed by the last time point (Fig. 4B) (see Table S6). Cohort three is defined by a single nonsynonymous mutation (Gly to Ser) that becomes fixed in a gene encoding a rod shape-determining protein.

10.1128/mBio.01969-16.9 TABLE S6  Polymorphism frequencies of *D. acidovorans* mutations. Download TABLE S6, XLSX file, 0.1 MB. Copyright © 2017 Olm et al. 2017 Olm et al. This content is distributed under the terms of the Creative Commons Attribution 4.0 International license.

By correcting for missing information due to lack of coverage at SNP sites in the ANG1 genome, we estimate that ~16 SNPs were fixed over the 1.5-year period. Thus, the rate of fixation is estimated as 10.1 SNPs/year. The remaining 150 SNPs that distinguish the SPH-1 genome sequenced in 2006 (S. Kjelleberg, personal communication) and ANG1 genomes likely arose between the time the SPH-1 genome was sequenced and the first metagenome time point (~6.5 years).

Given information about the SNP accumulation rate, we estimated how long ago the strain was separated from the original source culture. Assuming that the measured value approximates the rate of SNP formation over longer time periods, we calculated that the ANG1 genotype present in the soil was separated from the SPH-1 population 16.5 years ago.

We classified all 166 fixed SNPs that distinguish the SPH-1 and ANG1 genomes as synonymous, nonsynonymous, or intergenic (Fig. 4A). Overall, the *pN*/*pS* ratio is 0.98.

## DISCUSSION

We reconstructed a complete *D. acidovorans* genome from a soil metagenome. Recovery of a complete genome from soil is an extremely unusual achievement, given that assembly becomes more difficult as sample complexity increases. We infer that this occurred in the present study because the genome was relatively abundant (~1% of the total DNA) and the population was nearly clonal, avoiding assembly problems that arise because of strain variation. The lack of microheterogeneity is atypical of soil populations. This and the uncanny similarity to an isolate genome raised the possibility that the genome derived from a contaminant, a hypothesis that was confirmed by PCR-based testing. The extremely high similarity between the contaminant *D. acidovorans* populations and the *D. acidovorans* SPH-1 genome gives us high confidence that one derived from the other. In this specific case, we could determine the source of the contaminant DNA. Consequently, this research serves as a case study of microbial metagenomics-based forensics. Importantly, we showed that a contaminant clade could be identified through base-pair-by-base-pair analysis of variable sites in population genomic data sets collected over time. This made it possible to distinguish strains that were native to the samples in which they occurred from those that were the contaminant. This approach should be generally applicable in strain-tracking investigations so long as high-quality genomes can be recovered.

Metagenomics-derived genomes have only very rarely been used to identify and track strains. The closest example to our work was a study by Loman et al. (29) that targeted pathogenic *Escherichia coli* in fecal samples and associated it with disease. However, their methods were specifically designed for the clinical setting and generated a draft genome that was too incomplete and fragmented for use in accurate strain tracking and *in situ* evolutionary analysis. Long stretches of contiguous sequence are needed to identify indels and regions associated with horizontal gene transfer, and higher genome completeness leads to more accurate estimates of evolutionary distance.

During analysis of the anthrax attack, the crux of the analysis related to determining the similarity between the weaponized strain and the Ames Laboratory strain (30, 31). As we show here, high-resolution determination of strain relatedness is possible given comprehensive comparative genomic information about the bacterium of interest. Given a reasonable estimate of the mutation rate and information about the genomic similarity between the weaponized strain and the Ames Laboratory strain, it would be possible to estimate how long ago the cultures were separated.

Most short-term evolution experiments performed to determine mutation rates are carried out under laboratory conditions. These rates may differ significantly from rates that are applicable under conditions experienced by populations growing in natural systems. Because we had access to data sets archived over a 1.5-year period, we could estimate the rate of evolution of the contaminant population in the relevant environment (tubing) and use this calibration to approximate the length of time separating it from the previously sequenced isolate. Our calculated value (10.1 substitutions/year) is very close to previously reported values of evolving pathogen populations (9.6 substitutions/year) (7) and orders of magnitude greater than estimates of natural *E. coli* populations (32). Using our calculated value, we estimate that separation of ANG1 from the source population occurred 16 years ago. SPH-1 was sequenced in 2006, and ANG1 was sequenced in 2014; hence, the longest time that the contaminant ANG1 population could have been separated from the SPH-1 isolate is 9 years. This result is within a factor of 2 of the expected value, despite the possibility of large errors in mutation accumulation rates due to variations in growth rates and stress (33). Thus, we conclude that the comparative genomics approach used here can constrain the time of separation of two populations. From a technical perspective, it is worth noting that this detailed analysis was successful even in a background of soil.

We defined three distinct SNP cohorts at time points intermediate between the first and last metagenomic sequencing events, suggesting the existence of three genotypic variants. The fact that all 15 SNPs are fixed in the final ANG1 population is unexpected and inconsistent with separate coevolving populations. Further, the frequencies of SNPs in cohorts 1 and 3 undergo dramatic fluctuations in frequency (in some cases dropping below our detection level) rather than exhibit a simple trajectory toward fixation. We attribute both observations to extensive redistribution of SNPs through homologous recombination, a common process among closely related bacteria (34–36). The homogenization of population variation is consistent with recombination acting as a cohesive force, countering the diversifying SNP formation processes that otherwise could lead to speciation.

The original SPH-1 strain was isolated from municipal sewage sludge, an environment very different from the biofilm tubing environment in which the ANG1 strain was growing (37). The *pN*/*pS* ratio (the population-based equivalent to the *dN*/*dS* ratio [the ratio of nonsynonymous to synonymous evolutionary changes or substitutions]) of ~1, determined on the basis of a comparison of the isolate and the ANG1 population, is inconsistent with stabilizing selection. It could reflect minimal selective pressure or the combination of positive and negative selection. Most of the populations studied previously have values consistent with stabilizing selection (*dN*/*dS* ratios of ~0.1) because they are shaped by overall negative selection with some genes under positive selection (2, 38). Given this, we consider it more likely that the *D. acidovorans* population was experiencing a mixture of positive and negative selection rather than no selection. Positive selection is not surprising, given a population that is evolving and adapting to an environment different from that where it was isolated. An alternative explanation is that the nonsynonymous mutations have not yet had enough time to be selected against (39). The larger number of deletions (particularly of phage and transposon sequences) compared to insertions in the ANG1 relative to the SPH-1 genome is indicative of genome streamlining, consistent with its adaptation to a defined laboratory environment. Again, the observation of streamlining rather than genomic expansion is informative regarding the recent population history.

In conclusion, we show that detailed strain-resolved metagenomic studies can detect a specific organism of interest in very complex samples, provide evidence that the strain is not native to the environment from which the sample was collected, and constrain its recent history. We demonstrate this by using the example of a contaminant that was introduced during the laboratory handling of metagenomic samples, but the approach is far more broadly applicable. We used statistical analysis to link the contaminant genotypic group to its source and comparative genomics to uncover aspects of its recent evolutionary history. Because there was confirmation of many conclusions by independent methods, this work serves as a case study of strain-resolved forensic metagenomics.

## MATERIALS AND METHODS

### Sample collection.

Soil collection and DNA extraction were performed as reported previously (24) and briefly described here. Soil samples were collected (with permission under application no. 27790) from the Angelo Coast Range Reserve meadow (39°44′21.4″N, 123°37′51.0″W) and from a nearby ridge within the Eel Critical Zone Observatory. Approximately 1 kg of soil was removed with sterilized stainless steel hand trowels at each depth. Samples were immediately flash frozen in a mixture of dry ice and ethanol and placed on dry ice for transport to the lab. DNA was extracted with Mo Bio Laboratories PowerMax Soil DNA Isolation kits from 10 g of soil from each depth. We optimized the protocol for our samples to maximize the DNA yield while minimizing shearing; each sample was vortexed for only 1 min, followed by a 30-min heating step at 65°C with inversion every 10 min. We performed two elution steps of 5 ml each and precipitated the DNA with sodium acetate and glycogen, resuspending it in 100 µl of 10 mM Tris buffer.

### Metagenomic sequencing, assembly, and binning.

Metagenomic DNA was sequenced at the Joint Genome Institute (JGI). DNA was sheared to 800 bp with the Covaris LE220 (Covaris) and size selected with the Pippin Prep (Sage Science). The fragments were treated by end repair, A tailing, and ligation of Illumina compatible adapters (IDT, Inc.) with the KAPA-Illumina library creation kit (KAPA Biosystems). Paired-end reads of 250 bp were generated on an Illumina Hiseq2500 with sequencing depth enumerated in Table S1. Reads were trimmed with Sickle (40) and assembled with IDBA-UD (41). Resulting scaffolds >1 kb in length were annotated with Prodigal (42) to predict open reading frames (ORFs) by using default metagenomic settings. Annotated protein sequences were searched against the KEGG (43), UniRef100 (44), and UniProt databases with USEARCH (45). All matches with bit scores of >60 were saved, and reciprocal best hits with bit scores of >300 were also cataloged. We identified rRNA sequences with Infernal (46) by searching against databases from the SSU-Align package (47) and tRNAs with tRNAscan_SE (48). Genome binning was carried out with the online interface within ggKbase as described previously (49) (http://ggkbase.berkeley.edu/). This method takes into account the phylogenetic profile, GC content, and coverage information. The completeness of bacterial bins was evaluated on the basis of the presence or absence of single-copy genes. Shannon diversity calculations were performed as described previously (50) with assembled rps3 genes.

### Genome curation.

Once *D. acidovorans* scaffolds were binned, they were manually curated in order to close gaps between scaffolds. This was done within the Geneious software package version 8.1.5 (26). Project reads were first mapped back to the contigs with Bowtie 2 (51), and Geneious was used to extend the contigs. Contigs were next ordered and oriented by being mapped to the *D. acidovorans* SPH-1 reference genome with ABACAS (52). Areas of overlap between adjacent contigs were used to combine two contigs into one. To circularize the genome, reads were mapped directly to the reference genome. The reference genome was edited to be in agreement with the reads, and regions between contigs were filled in with corresponding pieces of the edited reference genome. Reads were finally mapped back to the circularized genome, and every base was manually inspected to ensure sequence read support. To catalog the differences between genomes, Mauve (53) was used to align the genomes and the alignment was manually inspected for mutations.

### Phylogenetic tree.

An alignment was generated with all of the rpS3 genes in the Angelo metagenomes, as well as previously published rpS3 sequences identified as similar to Angelo rpS3 sequences by BLAST. All rpS3 amino acid sequences longer than 180 amino acids were aligned with MUSCLE (54). The full alignments were stripped of columns containing 95% or more gaps. A maximum-likelihood phylogeny was inferred with RAxML (55) run with the PROTGAMMLG model of evolution. The RAxML interface included calculation of 100 bootstrap iterations (MRE [majority rules extended]-based bootstrapping criterion).

### PCR testing.

Primers amplifying randomly selected portions of the *D. acidovorans* genome were synthesized by IDT, Inc., to generate a 500-bp insert (forward, 5′GGGTTGCACCATTGGTATT; reverse, 5′GTCAGCGCCTTCTTTTCAA). Primers that amplify a 150-bp product of the 16S rRNA gene were also synthesized (forward, 5′GTGSTGCAYGGYTGTCGTCA; reverse, 5′ACGTCRTCCMCACCTTCCTC) (56). Pure *D. acidovorans* DNA was purchased from the DSMZ culture collection (DSMZ reference no. 14801). Reactions were performed with 5 PRIME MasterMix 50-µl reaction mixtures with 1 µl of each primer at 10 mM run in a ThermoCycler for 35 cycles with a melting temperature of 50°C and an extension time of 1 min 30 s. Both sets of primers and a no-primer control were run on (i) 0.05 ng of *D. acidovorans* DNA, (ii) 4.95 ng of original extracted soil DNA (sample 13_1_20cm_4), and (iii) both of the above combined into a single reaction mixture.

### Paired read insert length profiling.

Reads were mapped from each analyzed project to the *D. acidovorans* SPH-1 genome with Bowtie 2 (51). For comparison, reads from project 13_1_20cm_4 were also mapped to the second most abundant bacterial genome, that of a bacterium belonging to the phylum *Gemmatimonadetes*. The resulting .sam file was converted to a sorted .Bam file with SAMtools (57), and the insert size was profiled with Picard (58) (command, Java –jar Picard_tools/CollectInsertSizeMetrics.jar MINIMUM_PCT=0.4).

### Comparison of *D. acidovorans* strains.

To determine if soil and sediment metagenomes contained the *D. acidovorans* strain ANG1 genome, PileupProfile.py (source code available at https://github.com/banfieldlab/mattolm-public-scripts) Was used to calculate the ANI of reads mapping to the ANG1 genome. ANI was at least 99.9% in all cases where the median coverage was ≥2 (see Table S1). To compare strains present in 46 public metagenomic projects sequenced at the JGI that had been flagged as possibly containing *D. acidovorans* contamination, reads were mapped to the *D. acidovorans* SPH-1 genome with Bowtie 2 (51). Projects with at least 20% of the bases having 5× coverage were said to have significant *D. acidovorans* present and were analyzed in more detail. To compare the strains in each metagenome, the custom script ReadComparer.py was used (source code available at https://github.com/banfieldlab/mattolm-public-scripts). Briefly, the program first aligns reads from all projects to the same genome and compares the mutational patterns between projects to determine the relatedness of strains. VarScan (59) was used to create the input files (command, java -jar VarScan.v2.3.8.jar pileup2cns −min-coverage 3), and the script was run with the command, ReadComparer.py −min_breadth 0.2 −matrix −dend −smart_ignore. The similarity matrix was then plotted into a dendrogram with R, and clusters were determined and colored on the basis of manual inspection of the resulting dendrogram.

### Mutation identification and profiling.

A number of methods were used to identify differences between *D. acidovorans* strains SPH-1 and ANG1. Mutations were identified with breseq (60) mapping reads from project 13_1_20cm_4 to the *D. acidovorans* SPH-1 genome. To identify larger indels, *D. acidovorans* genomes were aligned with Mauve (53) and the alignment was manually inspected for differences. Finally, VarScan (59) was used on reads mapping from 13_1_20cm_4 to the *D. acidovorans* ANG1 genome. All called mutations were manually verified by inspection of the region with the software package Geneious (26).

To track the frequency of mutations among all projects of the contaminant clade, polymorpher2.py (available at https://github.com/banfieldlab/mattolm-public-scripts) was used to determine the frequency of each base at all of the positions identified. Variants that were <315 bp apart (a 1% chance of occurring assuming a random distribution of variants) were excluded from mutation rate calculations on the basis of the assumption that they were likely acquired in a recombination event. Positions were required to have some coverage in all projects, which excluded 4.4% of the variants from the analysis. Polymorphisms with a frequency of at least 50% at the first time point that became nearly fixed at the last time point (≤0.1%) were said to be evolving and were used in rate calculations (including a correction to account for variants without sufficient coverage for analysis). Full calculation details are available in Data Set S1.

10.1128/mBio.01969-16.3 DATA SET S1  Source code of analysis done with Python. Download DATA SET S1, TXT file, 0.2 MB. Copyright © 2017 Olm et al. 2017 Olm et al. This content is distributed under the terms of the Creative Commons Attribution 4.0 International license.

ORFs predicted by Prodigal were used to determine the total number of synonymous and nonsynonymous sites in the genome, as well as to classify each variant as synonymous, nonsynonymous, or intergenic. Genome-wide *pN*/*pS* ratios were calculated with the formula (number of nonsynonymous substitutions/number of nonsynonymous sites)/(number of synonymous substitutions/number of synonymous sites).

### Data availability.

Raw reads for all of the soil and sediment metagenomes in this study are available at the JGI Genome Portal (JGI Proposal ID, 1430). Accession numbers for specific samples are in Table S1. The source code of custom scripts used in data analysis (polymorpher2.py, PileupProfile.py, and ReadComparer.py) is available at https://github.com/banfieldlab/mattolm-public-scripts. The complete *D. acidovorans* ANG1 genome sequence is in the NCBI GenBank database under accession no. CP019171, BioProject no. PRJNA297196.
