# Supplementary figures and images for: The Source and Evolutionary History of a Microbial Contaminant Identified Through Soil Metagenomic Analysis
Source: mBio. 2017 Feb 21;8(1):e01969-16. doi: 10.1128/mBio.01969-16 (PMC5358914; doi:10.1128/mBio.01969-16)

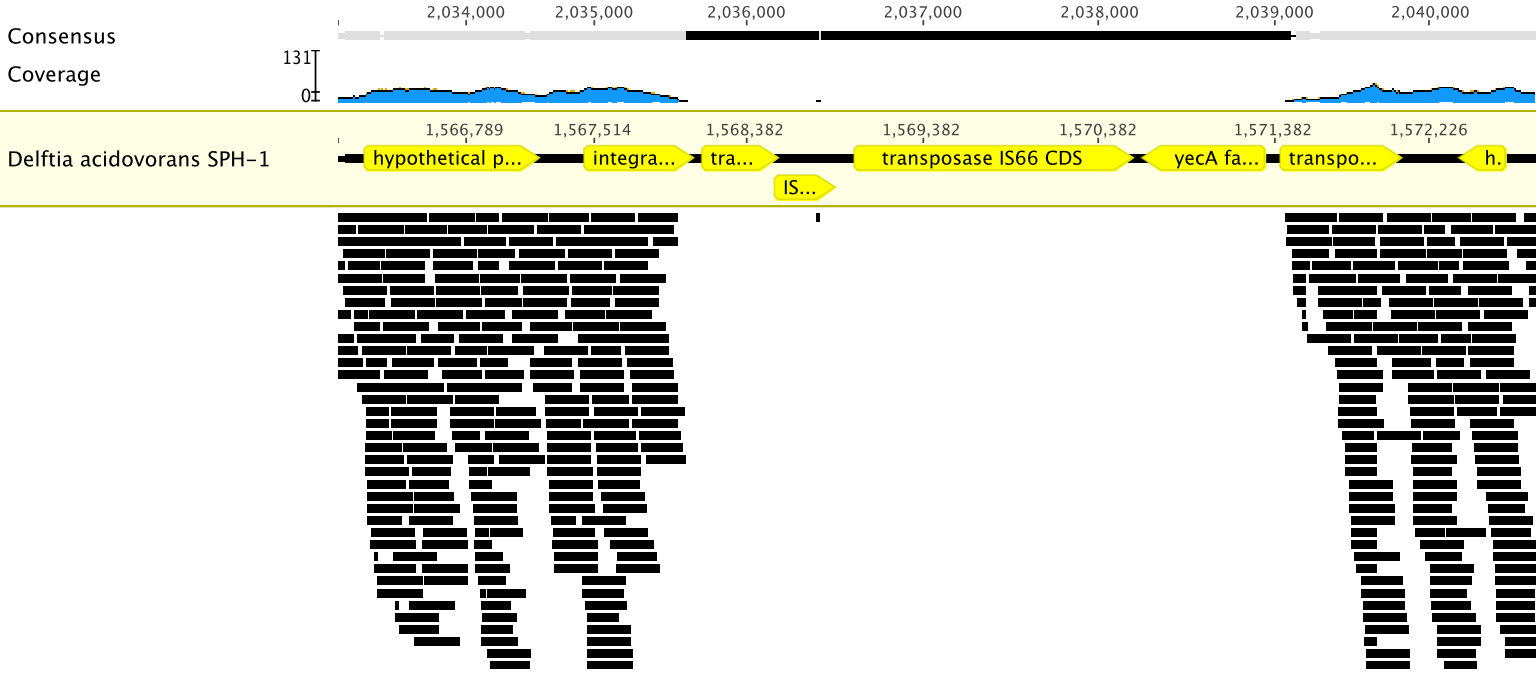

Supplement: FIG S1 [file mbo001173203sf1.pdf]

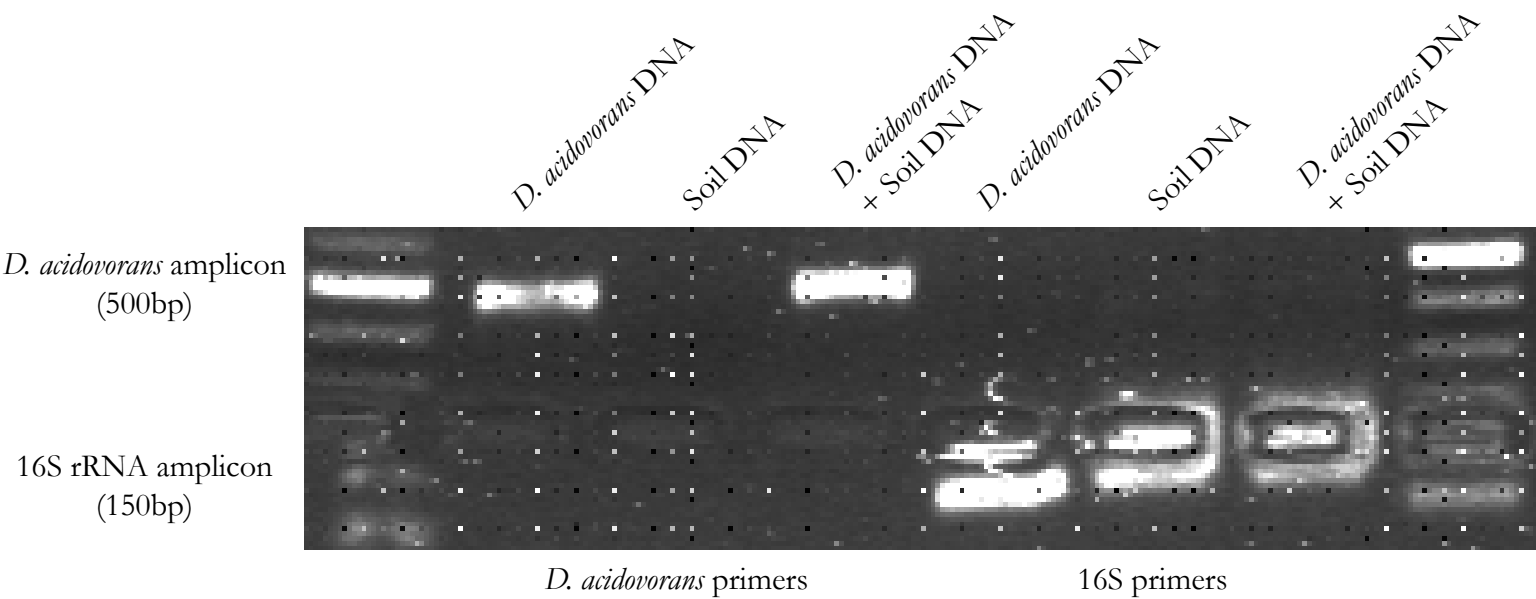

Supplement: FIG S2 [file mbo001173203sf2.pdf]
